# First-Principles Study on Periodic Pt_2_Fe Alloy Surface Models for Highly Efficient CO Poisoning Resistance

**DOI:** 10.3390/nano15151185

**Published:** 2025-08-01

**Authors:** Junmei Wang, Qingkun Tian, Harry E. Ruda, Li Chen, Maoyou Yang, Yujun Song

**Affiliations:** 1 Center for Modern Physics Technology, School of Mathematics and Physics, University of Science and Technology Beijing, Beijing 100083, China; 2 Shandong Key Laboratory of Optoelectronic Sensing Technologies/National-Local Joint Engineering Laboratory for Energy and Environment Fiber Smart Sensing Technologies, International School for Optoelectronic Engineering, Qilu University of Technology (Shandong Academy of Sciences), Jinan 250353, China; 3 Centre for Advanced Nanotechnology, University of Toronto, Toronto, ON M5S 3E3, Canada

**Keywords:** Pt_2_Fe alloy, low Pt contents, anti-CO poisoning, defects, d-band center

## Abstract

Surface and sub-surface atomic configurations are critical for catalysis as they host the active sites governing electrochemical processes. This study employs density functional theory (DFT) calculations and Monte Carlo simulations combined with the cluster-expansion approach to investigate atom distribution and Pt segregation in Pt-Fe alloys across varying Pt/Fe ratios. Our simulations reveal a strong tendency for Pt atoms to segregate to the surface layer while Fe atoms enrich the sub-surface region. Crucially, the calculations predict the stability of a periodic Pt_2_Fe alloy surface model, characterized by specific defect structures, at low platinum content and low annealing temperatures. Electronic structure analysis indicates that forming this Pt_2_Fe surface alloy lowers the d-band center of Pt atoms, weakening CO adsorption and thereby enhancing resistance to CO poisoning. Although defect-induced strains can modulate the d-band center, crystal orbital Hamilton population (COHP) analysis confirms that such strains generally strengthen Pt-CO interactions. Therefore, the theoretical design of Pt_2_Fe alloy surfaces and controlling defect density are predicted to be effective strategies for enhancing catalyst resistance to CO poisoning. This work highlights the advantages of periodic Pt_2_Fe surface models for anti-CO poisoning and provides computational guidance for designing efficient Pt-based electrocatalysts.

## 1. Introduction

High-performance Pt electrodes are crucial for the advancement of economical and efficient fuel cell technology [1,2,3]. Alloying Pt with transition metals has shown significant advantages in enhancing the catalytic activity and stability due to synergistic effects [4,5,6,7,8]. As one of the most abundant and cost-effective transition metals on earth, Fe alloy with Pt exhibits high catalytic activity while reducing the amount of Pt [9,10]. The surface structure and composition of Pt-Fe alloys determine the number of active sites and the adsorption energy of intermediates, thereby optimizing catalytic performance [11,12,13]. However, realistic Pt-based alloys often exhibit disordered structures, with active atoms distributed stochastically in three dimensions and diverse surface compositions. This disorder is computationally predicted and experimentally observed to facilitate dissolution of transition metals from the surface, leading to rapid performance degradation under fuel cell operating conditions [14,15,16].

Many strategies, guided by both theory and experiment, have been explored to regulate surface structure and improve catalytic activity [3,17,18]. For example, ordered intermetallic phases like L1_0_-PtFe, L1_2_-Fe_3_Pt and L1_2_-PtFe_3_ have been theoretically investigated and experimentally synthesized to stabilize surface transition metals and modulate reaction barriers [19,20,21,22,23]. Compared to disordered surfaces, the stronger lattice strain experienced by surface Pt in these ordered compounds significantly alters the d-band center and consequently the adsorption energy of key intermediates [23,24,25,26]. Zhao et al. suggested that Pd substitution could facilitate the formation of L1_2_-Pt_3_Fe intermetallic at lower annealing temperatures, resulting in a downward shift of the d-band center and improved alkaline hydrogen oxidation reaction performance [27]. Similarly, Gong et al. demonstrated that compression strains introduced by Pt-skin surfaces, formed via Pt segregation and Fe core dissolution, modify the d-band electronic structure of surface Pt and the associated reaction barrier [28]. Therefore, optimizing the performance of Pt-Fe alloys through surface structural modification is highly effective [29]. A thorough understanding of surface segregation and structural evolution mechanisms is essential for this rational design.

Previous theoretical research on the catalytic activity of Pt alloys often relied on constructing simplified hypothetical surface models by manually varying the atomic composition within limited unit cells [30]. While high-resolution transmission electron microscopy provides valuable experimental insights into surface structures, the inherent complexity and dynamism of surfaces mean that observed structures might represent snapshots or averages, and computational models often need refinement to capture the full configurational space [15,20]. Crucially, the vast number of possible atomic configurations makes exhaustive sampling using first-principles calculations alone computationally prohibitive. Consequently, computationally predicting the thermodynamically stable surface structures and their evolution under realistic conditions remains a significant challenge.

In this work, we employed a cluster expansion (CE) approach combined with density functional theory (DFT) calculation to simulate the energy configuration dependence of Pt-Fe alloy and identify the lowest energy configurations for each component. By further utilizing Monte Carlo (MC) simulations, we efficiently explore the vast configurational space and predict the equilibrium Pt distribution across surface and sub-surface layers as a function of overall Pt concentration and simulated annealing temperature. Our calculations reveal the thermodynamic stability of a periodic Pt_2_Fe surface alloy phase, incorporating specific defect structures, particularly at low Pt content and low temperatures. Notably, the predicted formation of this Pt_2_Fe surface phase aligns with trends observed in related experimental systems [12]. Electronic structure analysis within our DFT framework demonstrates that the formation of this Pt_2_Fe surface alloy lowers the d-band center of Pt atoms, weakening CO adsorption and thereby enhancing resistance to CO poisoning. However, the strains introduced by defects within this phase lead to a nonlinear relationship between d-band center and CO adsorption energy. Detailed analysis via density of states (DOS) and crystal orbital Hamilton population (COHP) confirms that these defect-induced strains generally strengthen Pt-CO interactions, despite sometimes modulating the d-band center. This research establishes a computationally efficient and predictive framework based on CE-DFT-MC for theoretically exploring surface segregation and designing binary alloy catalysts. The approach is readily extendable to more complex systems and microstructure optimization.

## 2. Calculation Methods

**Figure 1:**
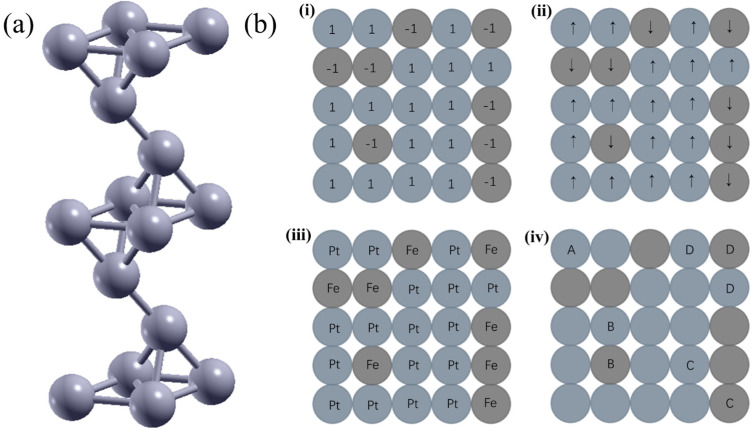
(**a**) The basic unit structure used in the calculation; (**b**) two-dimensional Pt-Fe simple square lattice diagrams used in the CE method. (**i**) $\sigma_{p}$ values; (**ii**) spins in Ising model, up arrow means $\sigma_{p}=+1$, down arrow means $\sigma_{p}=-1$; (**iii**) atomic elements; (**iv**) schematic of representative clusters.

The DFT calculations were performed using Quantum ESPRESSO (QE, v7.0) with the Perdew–Burke–Ernzerhof (PBE) generalized gradient approximation (GGA). The interaction between ions and electrons was described using the projector augmented wave (PAW) method. Also, the plane wave basis sets were employed with an energy cut-off of 100 Ry. A seven-layer model of Pt_x_Fe_y_ was built for face-centered cubic (FCC) (111) surfaces separated by a 15 Å vacuum layer on the z-axis, as shown in Figure 1a. While bulk Fe is typically body-centered cubic (BCC) and Pt is FCC at room temperature, the literature indicates that FCC Fe can be achieved in thin films [31]. Therefore, this study is based on the scenario where both Fe and Pt exhibit the FCC lattice structure. The electron charge density was sampled using the Monkhorst–Pack special k-point method in the Brillouin zone, with the number of k-points per reciprocal atom exceeding 8000. In structural optimizations, the residual forces among atoms converged below $10^{-3}\;Ry/Bohr\;\left(\sim 0.025\;eV/Å\right)$. The self-consistent convergence accuracy was $10^{-8}\;Ry$. All calculations incorporated spin polarization effects, with both ferromagnetic (FM) and antiferromagnetic (AFM) configurations evaluated. The basic unit structure is shown in Figure 1a. The Local-Orbital Basis Suite Towards Electronic-Structure Reconstruction (LOBSTER, v5.1.1) software package was used to calculate the DOS and the COHP [32].

The CE method is a parameterized model for rapidly and accurately predicting the energies of various atomic configurations on a crystal lattice [33,34]. Consider a crystalline system with N lattice sites. The occupation state of each site p is described by a discrete variable $\sigma_{p}$. In multicomponent systems where the number of components M = 2m or M = 2m + 1, $\sigma_{p}$ can take values $\pm m,\;\pm \left(m-1\right),\ldots ,\pm 1$ and 0. Any configuration of this N-site crystal can be represented by an N-dimensional vector $\sigma =\{\sigma_{1},\;\sigma_{2},\ldots ,\sigma_{N}\}$. For arbitrary functions $f\left(\sigma \right)$ and $g\left(\sigma \right)$, their scalar product is defined as:

(1) $$\left\langle f,g\right\rangle =\rho_{N}^{0}{Tr}^{\left(N\right)}\left[f\cdot g\right]$$

Here, ${Tr}^{\left(N\right)}$ denotes the matrix trace operation, and $\rho_{N}^{0}=M^{-N}$ is a normalization constant. An orthogonal basis is constructed using Chebyshev polynomials of the first kind. For any lattice site p, we define M orthogonal polynomials:

(2) $$\Theta_{2s}\left(\sigma_{p}\right)=\sum_{k=0}^{s}c_{k}^{\left(s\right)}\sigma_{p}^{2k},s=0,1,\ldots ,m-1,\left(m\right)$$

(3) $$\Theta_{2s+1}\left(\sigma_{p}\right)=\sum_{k=0}^{s}d_{k}^{\left(s\right)}\sigma_{p}^{2k+1},s=0,1,\ldots ,m-1$$

The coefficients $c_{k}^{\left(s\right)}$ and $d_{k}^{\left(s\right)}$ are determined by the orthogonality relation:

(4) $$\begin{array}{c} \langle \Theta_{n}\left(\sigma_{p}\right),\Theta_{n^{’}}\left(\sigma_{p}\right)\rangle =\rho_{N}^{0}{Tr}^{\left(N\right)}\Theta_{n}\left(\sigma_{p}\right)\Theta_{n^{’}}\left(\sigma_{p}\right)\\ =M^{-1}\sum_{\sigma_{p}=-m}^{m}\Theta_{n}\left(\sigma_{p}\right)\Theta_{n^{’}}\left(\sigma_{p}\right)=\delta_{nn^{’}} \end{array}$$

where $\delta_{nn^{’}}$ is the Kronecker delta function. For any two distinct lattice points p and $p^{’}$, and for n≥1, $n^{’}\geq 1$, Equation (4) is generalized as:

(5) $$\left\langle \Theta_{n}\left(\sigma_{p}\right),\Theta_{n^{’}}\left(\sigma_{p^{’}}\right)\right\rangle =M^{-2}\sum_{\sigma_{p}=-m}^{m}\sum_{\sigma_{p^{’}}=-m}^{m}\Theta_{n}\left(\sigma_{p}\right)\Theta_{n^{’}}\left(\sigma_{p^{’}}\right)=\delta_{nn^{’}}\delta_{pp^{’}}$$

Additionally, the following completeness relation holds:

(6) $$\sum_{n=0}^{M-1}\Theta_{n}\left(\sigma \right)\Theta_{n^{’}}\left(\sigma^{’}\right)=M\delta_{\sigma \sigma^{’}}$$

By forming products of $\Theta_{n}\left(\sigma_{p}\right)$ values over all possible combinations of polynomial orders n and lattice points p, a complete set of orthogonal cluster functions $\phi_{\alpha s}$ is obtained. For a cluster of sites $\alpha =\{p,p^{’},\ldots ,p^{’’}\}$ and index set $s=\{n,n^{’},\ldots ,n^{’’}\}$, the cluster function is defined as:

(7) $$\phi_{\alpha s}\left(\sigma_{p},\sigma_{p^{’}},\ldots ,\sigma_{p^{’’}}\right)=\Theta_{n}\left(\sigma_{p}\right)\Theta_{n^{’}}\left(\sigma_{p^{’}}\right)\cdots \Theta_{n^{’’}}\left(\sigma_{p^{’’}}\right)$$

Orthogonality follows from Equation (5):

(8) $$\left\langle \phi_{\alpha s},\phi_{{\beta s}^{’}}\right\rangle =\delta_{\alpha \beta }\delta_{ss^{’}}$$

The completeness relation generalizes to:

(9) $$\rho_{\alpha }^{0}\sum_{s}\phi_{\alpha s}\left(\sigma \right)\phi_{\alpha s}\left(\sigma^{’}\right)=\delta_{\sigma \sigma^{’}}$$

where $\rho_{\alpha }^{0}=M^{-n_{\alpha }}$ and $n_{\alpha }$ is the number of sites in cluster α.

Any configuration-dependent function expands as:

(10) $$f\left(\sigma \right)=\sum_{\alpha s}\;f_{\alpha s}\phi_{\alpha s}\left(\sigma \right)$$

with coefficients given by:

(11) $$f_{\alpha s}=\left\langle \phi_{\alpha s},\;f\right\rangle$$

Extracting the constant term $f_{0}=\langle \phi_{0},\;f\rangle$ (empty cluster):

(12) $$f\left(\sigma \right)=f_{0}+\sum_{\alpha s}\;f_{\alpha s}\phi_{\alpha s}\left(\sigma \right)$$

where the summation includes only non-empty clusters. The coefficients $f_{\alpha s}$ are termed effective cluster interactions (ECIs). Equation (12) is exact when all clusters are included, but practical implementations use distance-based truncation to balance accuracy and computational efficiency.

Alloy Theoretic Automated Toolkit (ATAT, v3.50) is the software used in the CE method [35]. A two-dimensional Pt-Fe simple square lattice used in cluster expansion schematically is shown in Figure 1b. Each lattice site *p* is assigned to a site-occupation variable $\sigma_{p}$, where $\sigma_{p}=-1$ corresponds to an Fe atom occupying the site and $\sigma_{p}=+1$ indicates Pt occupation, which are shown in Figure 1b(i–iii). Representative clusters $\phi_{\alpha s}$ are depicted in Figure 1b(iv): (A) single-site cluster ($n_{\alpha }$ = 1), (B) nearest-neighbor pair cluster ($n_{\alpha }$ = 2), (C) next-nearest-neighbor pair cluster ($n_{\alpha }$ = 2) and (D) nearest-neighbor triplet cluster ($n_{\alpha }$ = 3). Initially, we generated FePt structures with up to 63 atoms and incorporated their DFT calculation results as the training data. Clusters of atoms with maximum atomic spacing of 12 Å for pair clusters, 7 Å for triplet clusters and 5 Å for quadruplet clusters were considered. Leave-one-out cross-validation (LOOCV) was used to obtain the ECIs.

## 3. Results and Discussion

**Figure 2:**
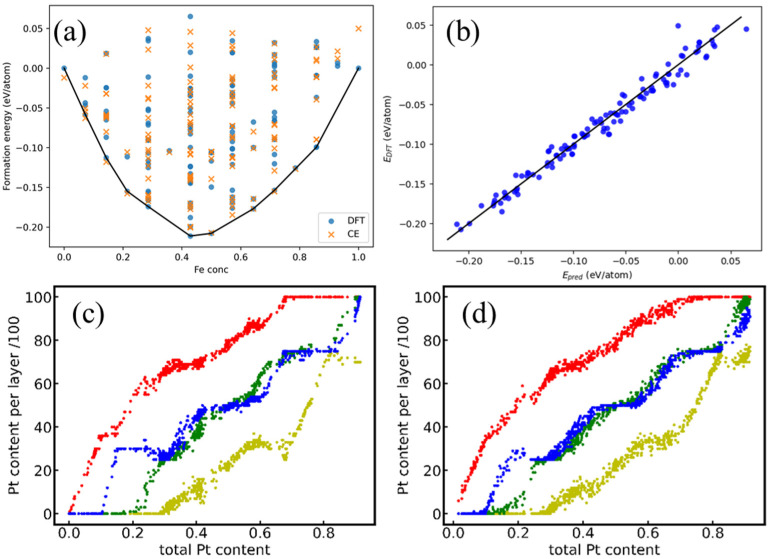
(**a**) The energy maps obtained from DFT calculation (blue dots) and predicted by cluster expansion (orange crosses); (**b**) predicted energies using the CE approach versus energies obtained using DFT calculations; (**c**,**d**) the distribution of Pt atoms in each layer after annealing temperature of approximately 350 °C (**c**) and 650 °C (**d**). Red dots represent the first surface layer, yellow dots represent the second layer, green dots represent the third layer, blue dots represent the fourth layer.

**Figure A1:**
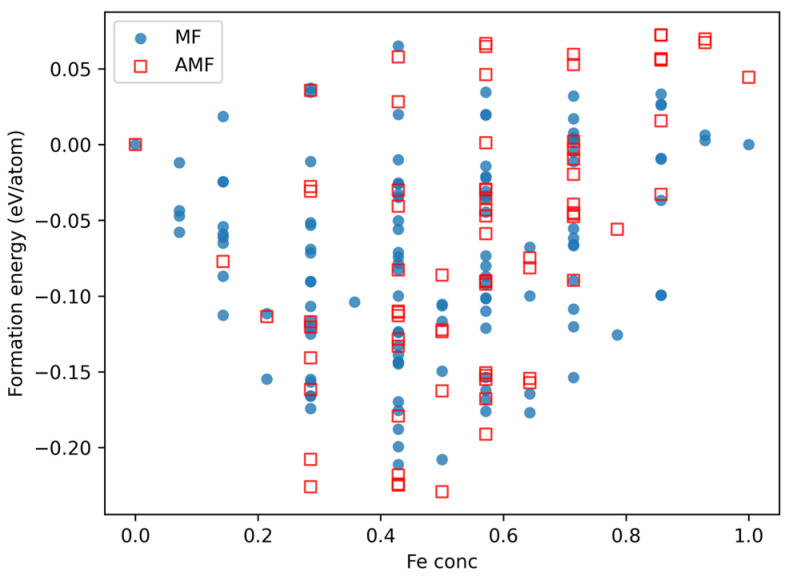
Energy maps of FM and AFM states in Pt-Fe systems calculated by DFT. For part of Pt-rich configurations with negligible FM-AFM energy differences, only FM energies are displayed.

To model the complex configurational energy landscape of Pt-Fe alloys, a CE Hamiltonian was parametrized using a training dataset comprising 119 distinct atomic configurations, whose energies were obtained from DFT calculations. The comparison of FM and AFM data for these configurations is presented in Figure A1, revealing that FM is generally more stable than AFM in the low Pt region (Pt concentration < 0.3). The CE parameters were optimized to minimize the LOOCV error. Figure 2a compares the FM DFT-calculated formation energies (blue dots) with those predicted by the fitted CE model (orange crosses) for all structures in the dataset. The excellent agreement is quantified by a LOOCV score of 12.9 meV/atom and a root mean square error (RMSE) of 10.2 meV/atom (Figure 2b). These low error metrics confirm the high predictive accuracy of the CE Hamiltonian across the compositional and configurational space explored, validating its use for subsequent Monte Carlo simulations.

To elucidate the thermodynamic evolution of surface structure and composition under annealing conditions, semi-grand-canonical Monte Carlo simulations [36] were performed. Simulations were conducted at represented temperatures of approximately 350 °C and 650 °C using systems containing 700 to 54,208 atoms, with atomic compositions in each layer of the slab recorded (Figure 2c,d). Due to structural symmetry between top and bottom surfaces, atomic concentrations in layers 1/7, 2/6 and 3/5 are essentially identical; thus, only layers 1–4 are presented. Each simulation ran for over 100,000 steps. Convergence to equilibrium was assessed by monitoring the fluctuations in layer concentrations and total system energy. The criterion for equilibrium was met when the differences in these fluctuations between two consecutive blocks of 10,000 steps were less than 3%. The MC simulations consistently revealed a strong thermodynamic driving force for Pt surface segregation across the simulated temperatures and composition. As illustrated in Figure 2c,d, Pt atoms preferentially occupy the surface layers (layer 1 and 7), while Fe atoms enrich the sub-surface layers (layer 2 and 6), resulting in a characteristic gradient composition profile. This segregation behavior is primarily attributed to the lower surface segregation energy of Pt compared to Fe and the associated reduction in system free energy, confirming that Pt segregation occurs spontaneously under vacuum conditions without external influences [37]. This Pt-rich surface is computationally predicted to enhance catalyst active site availability, while the Fe-enriched sub-surface contributes to reduced material cost. Notably, the Pt concentration profiles in the third and fourth layers generally exhibited close similarity. The slight difference can be attributed to the fact that the third layer is superficial compared with the fourth layer.

**Figure A2:**
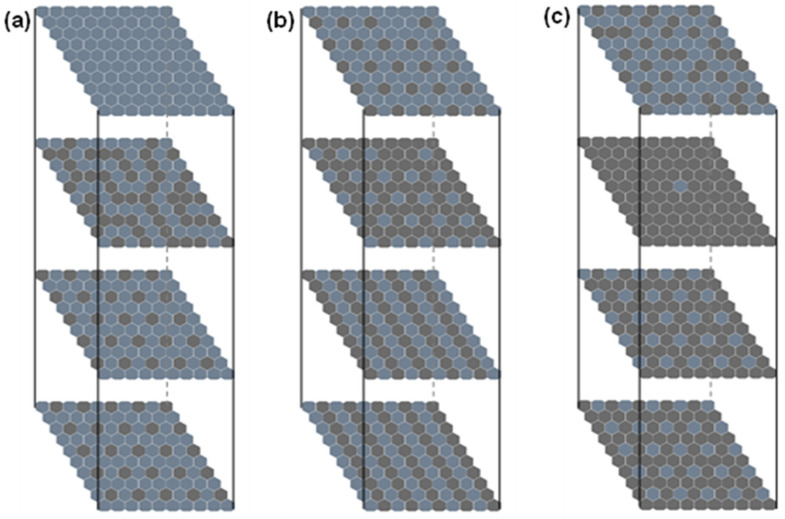
The slab structure of surface four layers at the overall Pt content of (**a**) 0.75, (**b**) 0.5 and (**c**) 0.29; the blue is Pt and the gray is Fe.

At the lower simulated annealing temperature of 350 °C, the MC simulations predicted the formation of distinct, stable surface and near-surface phases. This is evidenced by the pronounced horizontal steps in the Pt concentration profile (Figure 2c) occurring at approximately 75%, 50%, 29% and 20% overall Pt content. Representative atomic configurations extracted from the MC trajectories near the 75%, 50% and 29% Pt levels are shown in Figure A2a–c. The Pt content consistently follows surface layer > layer 3 ≈ layer 4 > sub-surface layer (layer 2). Crucially, the atomic arrangements within Layer 3 and Layer 4 of these representative configurations correspond to well-known ordered intermetallic phases: Fe_3_Pt (L1_2_ structure), FePt (L1_0_ structure) and FePt_3_ (L1_2_ structure). The formation of these specific ordered phases (Fe_3_Pt, FePt, FePt_3_) within the central layers of the slab model is consistent with the equilibrium bulk phases expected from the Pt-Fe phase diagram at these compositions. This demonstrates that the central layers (Layers 3, 4) of the seven-layer slab model effectively mimic the structural behavior of the bulk material under the simulated conditions, providing a robust foundation for modeling the surface region (Layers 1–2). Notably, when the overall Pt concentration falls between 15% and 25%, a sharp redistribution of Pt atoms occurs between the third and fourth layers (Figure 2c). As the overall Pt concentration approaches ~25%, the Pt content in the third layer plummets sharply from ~25% to nearly 0%, while simultaneously the Pt content in the fourth layer surges from ~25% to ~30%. 

**Figure 3:**
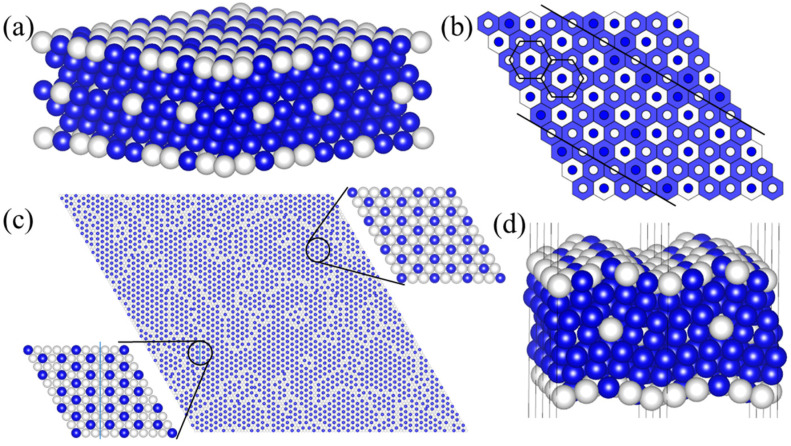
(**a**) The structure at the Pt atom ratio of 24% with white and blue representing Pt and Fe atoms, respectively; (**b**) the corresponding surface (sphere) and core layer structure (hexagon)—the white balls represent Pt atoms and the blue balls represent Fe atoms; (**c**) the surface atom distribution at the Pt atom ratio of 24%; (**d**) the Pt_2_Fe surface structure after DFT relaxation.

**Figure A3:**
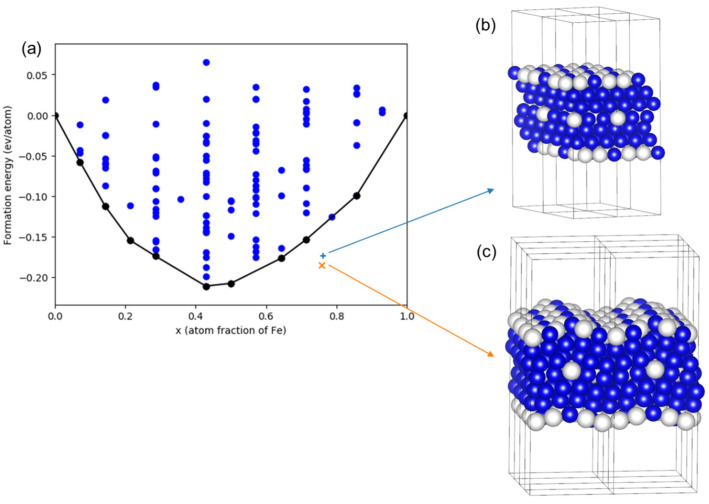
(**a**) The energy maps obtained from DFT calculation (blue ball), and the ground state on convex hull (black ball); (**b**) the defect-free Pt_2_Fe surface structure after DFT relaxation; (**c**) the Pt_2_Fe surface structure after DFT relaxation—the white balls represent Pt atoms, the blue balls represent Fe atoms.

A representative atomic configuration extracted from MC simulations at ~24% overall Pt content (Figure 3a) illustrates that Pt atoms originally in the third layer aggregate and transfer towards the fourth layer, facilitating the formation of a new Fe_2_Pt-like phase within the fourth layer. Consequently, the third layer becomes almost entirely depleted of Pt and occupied by Fe atoms. Crucially, concurrent with this subsurface restructuring, the MC simulations predict the development of a distinctive periodic Pt_2_Fe alloy phase within the critical surface layer, as depicted in Figure 3b. This Pt_2_Fe surface phase is consistently accompanied by the formation of surface defects in the simulations. To gain deeper insight into the nature of these surface defects, we performed large-scale MC simulations involving 54,208 atoms. The resulting mesoscale configuration (Figure 3c) clearly shows the spontaneous emergence of irregular defect structures distributed across the surface. To assess the stability and local structural impact of these defects, we performed DFT structural relaxation on the defective Pt_2_Fe surface model derived from Figure 3a, conducting calculations for both FM and AFM magnetic orderings. FM configurations were more stable by 0.1609 eV/atom than AFM and thus identified as the optimal magnetic state. The relaxation (Figure 3d) confirms that the defects induce significant localized lattice distortion. Calculations of the defect formation energy (Figure A3) further affirm the thermodynamic stability of these distorted configurations, supporting their persistence under the simulated conditions.

Comparison of the Pt concentration profiles annealed under 350 °C and 650 °C (Figure 2c,d) provides key insights into the thermal stability of the Pt_2_Fe surface phase: The distinct plateau region centered around ~20% Pt content observed at 350 °C signifies the stable formation and persistence of the Pt_2_Fe surface phase over a range of compositions at this lower temperature. In contrast, at the higher simulated temperature of 650 °C, this plateau region vanishes and is replaced by a significantly broader dispersion in Pt content across the surface layer. This dispersion strongly suggests that elevated temperatures promote the introduction of numerous point defects (e.g., vacancies, anti-sites) and compositional fluctuations within the Pt_2_Fe surface phase, disrupting its long-range periodicity. Importantly, this computational prediction of enhanced Pt_2_Fe surface phase stability at lower annealing temperatures aligns with the general trend observed in related experimental studies of Pt-Fe catalysts, where improved CO tolerance is often reported for PtFe catalysts annealed under the low temperature [12].

**Figure 4:**
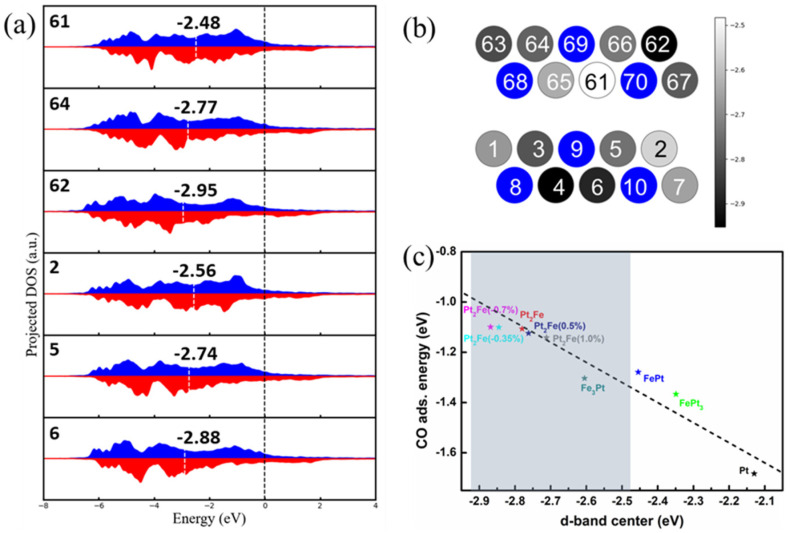
(**a**) The d-band pDOS of Pt in the upper and bottom surfaces, blue and red correspond to spin up and spin down, respectively; (**b**) the d-band center of Pt in the upper and bottom surfaces, where color bar indicates d-band center values, atomic labels correspond to (**a**) and blue spheres represent Fe atoms; (**c**) the d-band center and CO adsorption energy—the shaded area is for the upper and lower surface Pt atoms.

The d-band projected DOS (pDOS) of Pt atoms in structures containing the defective Pt_2_Fe surface phase (Figure 3d) was investigated, as shown in Figure 4a. The results indicate that the d-band of Pt atoms remains relatively unchanged overall, only contracting or expanding in response to stress from defects. This contraction or expansion results in a shift of the d-band center upward or downward, respectively, due to the high overall d-band filling of Pt. The d-band center $\epsilon_{d}$ was calculated using the Equation (13) below,

(13) $$\epsilon_{d}=\frac{\int_{-\infty }^{E_{F}}\epsilon \rho_{d}\left(\epsilon \right)d\epsilon }{\int_{-\infty }^{E_{F}}\rho_{d}\left(\epsilon \right)d\epsilon }$$

where *E_F_* denotes the Fermi level, ϵ is the d-orbital energy, $\rho_{d}$ is the corresponding density of states. The d-band centers for Pt atoms on the upper and lower surfaces range from −2.48 eV to −2.95 eV (Figure 4b), all lower than those calculated for FePt and FePt_3_, and some even lower than Fe_3_Pt (Figure 4c). Studies on CO adsorption show that the CO adsorption energy weakens with the d-band center downwards, corroborating previous reports [38]. Therefore, the Pt_2_Fe surface phase with a low d-band center should inherently exhibit enhanced resistance to CO poisoning.

**Figure A4:**
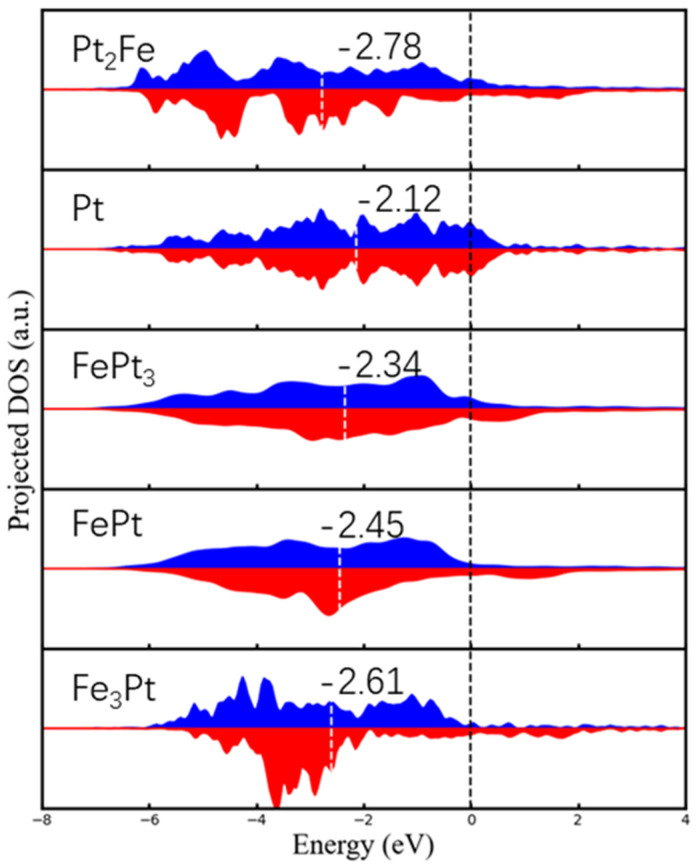
The d-band pDOS of surface Pt for Pt-Fe slab at different Pt/Fe atom ratios. The blue areas and the red areas correspond to the spin up cases and the spin down cases, respectively.

To isolate the intrinsic electronic effect of the Pt_2_Fe surface phase from defect-induced perturbations, we calculated the electronic structure and CO adsorption energy for a perfectly ordered, defect-free Pt_2_Fe surface model. Our calculations indicate ferromagnetic ordering is energetically favored over antiferromagnetic by 0.1295 eV/atom; thus, all subsequent computations adopted the ferromagnetic state. As shown in Figure 4c and Figure A4, the defect-free Pt_2_Fe surface phase possesses the lowest Pt d-band center among all investigated structures (Fe_3_Pt, PtFe_3_, PtFe, pure Pt), confirming that the ordered Pt_2_Fe stoichiometry itself, likely due to the increased number of Fe nearest neighbors surrounding surface Pt atoms, intrinsically lowers the d-band center. Based on the lattice constants from the relaxed structure and the defect-free Pt_2_Fe phase structure calculated in Figure 3, we applied a series of strain values (1%, 0.5%, −0.35% and −0.7%) to the defect-free Pt_2_Fe surface structures, where positive values represent tensile strain and negative values represent compressive strain. Application of 0.5% and 1% tensile strain caused the d-band center to upshift from −2.78 eV (strain-free) to −2.76 eV and −2.71 eV, respectively (Figure 4c). Concomitantly, the calculated CO adsorption energy strengthened (became more negative), increasing from −1.11 eV to −1.12 eV and −1.14 eV. Notably, most literature reports indicate that CO adsorption energies on oxygen-free Pt catalysts are predominantly below −1.5 eV [39,40]. While our calculated value is near −1.1 eV, implying the markedly enhanced resistance to CO poisoning. This result clearly demonstrates an approximate linear relationship under tensile strain: the d-band center upshift leads to a stronger CO adsorption. Conversely, applying 0.35% and 0.7% compressive strains downshifted the d-band center to −2.85 eV and −2.87 eV, respectively. However, the corresponding CO adsorption energies (−1.10 eV and −1.10 eV) did not weaken as might be linearly extrapolated from the tensile case. Instead, they remained nearly constant or slightly weakened relative to the strain-free value (−1.11 eV). This result on the strain dependent CO adsorption energies indicates a significant deviation from the simple linear d-band center model under the compressive strain.

**Figure 5:**
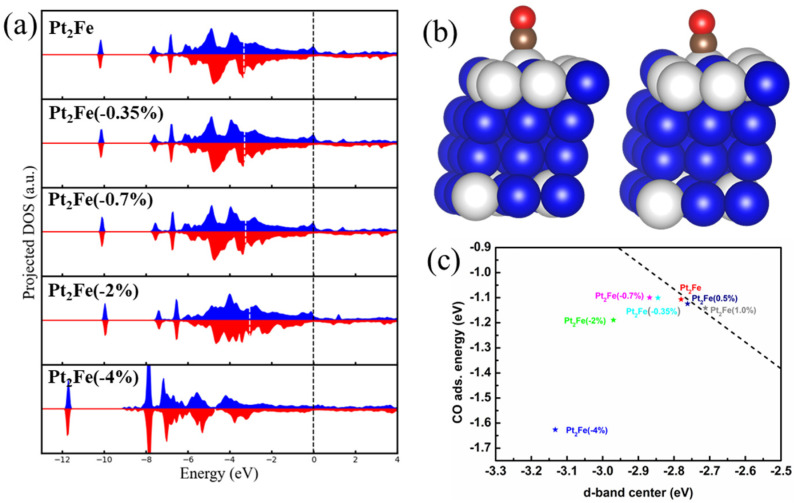
(**a**) The d-band pDOS of structures with Pt_2_Fe surface under different compressive strains, blue and red correspond to spin up and spin down, respectively; (**b**) the structure under 2% and 4% compressive strains—the white atom is Pt, the blue atom is Fe, the brown atom is C and the red atom is O; (**c**) the relationship between d-band center and CO adsorption energy of different structures.

To elucidate the origin of the nonlinear CO adsorption behavior under compressive strain, we performed additional DFT calculations by applying larger compressive strains (2%, 4%). The Pt d-band pDOS in Figure 5a shows a pronounced downward shift of the bonding states under 4% compression. The geometry structures shown in Figure 5b indicate that the adsorbed Pt site tends to be pulled out except under the extreme 4% strain. In addition, calculated CO adsorption energies consistently strengthened (became more negative) with increasing compressive strain magnitude, further deviating from the linear trend established under the tensile strain, as shown in Figure 5c.

Integrating these results, a volcano plot relationship is observed between the d-band center and CO adsorption energy. When the d-band center is higher (above Pt_2_Fe), the energy difference between the orbitals in the Pt atom that are close to the symmetry of CO orbitals is larger, resulting in lower bonding strength. And the main effect on the adsorption energy is the electron transfer caused by the overall upward shift of the Pt d-orbitals. Consequently, an upward d-band center shift enhances adsorption energy. Conversely, when the d-band center is lower (below Pt_2_Fe), the energy difference between the orbitals in Pt atoms that are close to the symmetry of CO orbitals narrows, and the bonding between them contributes significantly to the adsorption energy. In this regime, a downward d-band center shift enhances adsorption energy. Therefore, within the Pt-Fe system, the proposed periodic Pt_2_Fe surface structure exhibits a theoretically low CO adsorption energy approaching the volcano peak. Moreover, this analytical framework can be extended to other systems, facilitating the theoretical analysis of catalytic mechanisms and aiding in the design of enhanced catalysts.

**Figure 6:**
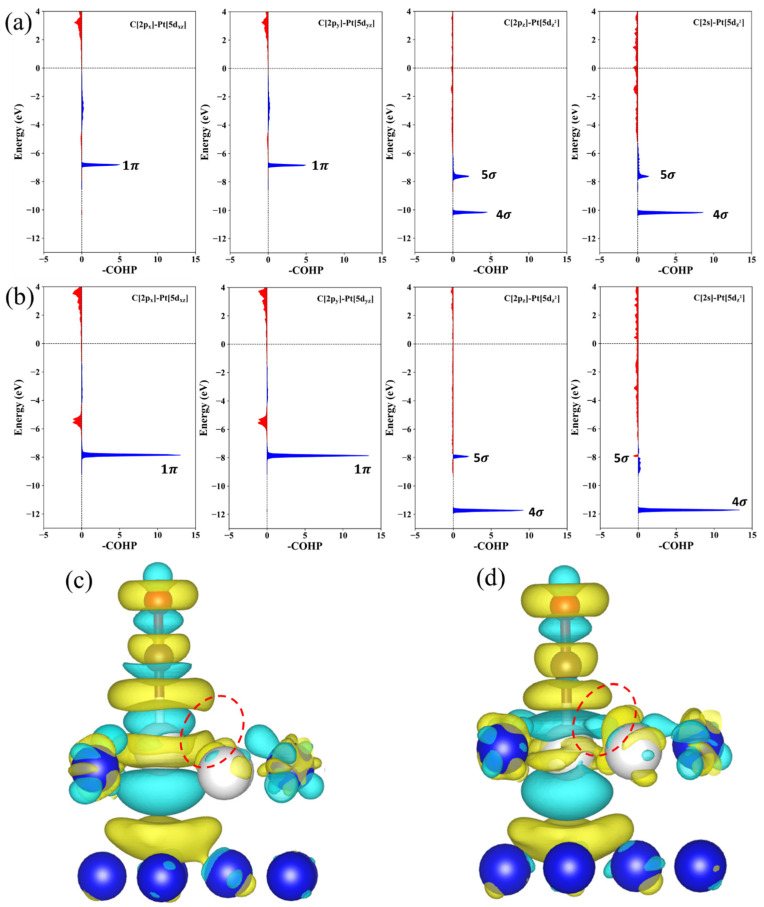
(**a**) The COHP of Pt_2_Fe, the blue area denotes bonding interactions, red represents anti-bonding interactions; (**b**) the COHP of Pt_2_Fe under 4% compressive strain; (**c**) the differential charge density of Pt_2_Fe adsorbed CO structure; (**d**) the differential charge density of Pt_2_Fe under 4% compressive strain with CO adsorption. Yellow and blue isosurfaces indicate electron accumulation and depletion, respectively.

To gain a deep mechanistic insight into the Pt-CO bonding interactions, particularly under strains, we performed COHP analysis based on DFT calculations. COHP decomposes the band structure energy into bonding, non-bonding and anti-bonding contributions between specific atom pairs. For consistency with common practice (and crystal orbital overlap population convention), the negative of the COHP (-COHP) is plotted, where peaks in the positive direction indicate net bonding interactions, and peaks in the negative direction indicate net anti-bonding interactions (Figure 6a,b). Analysis of the Pt-CO interactions on the Pt_2_Fe surface phase reveals that the dominant bonding contributions arise from interactions between the d-orbitals of surface Pt atoms and the frontier molecular orbitals of CO, specifically the 4σ, 5σ and 1π orbitals. These interactions are significant due to favorable energy alignment and symmetry matching. In contrast, interactions involving other CO orbitals (3σ, 2π*, 6σ) are negligible due to large energy mismatches with the Pt d-states; these insignificant interactions are omitted from Figure 6. Crucially, comparison of the -COHP profiles for the unstrained case (Figure 6a) and the system under 4% compressive strain (Figure 6b) demonstrates a key effect of large compression: The bonding states associated with the Pt-C interaction undergo a pronounced downward shift in energy under 4% compressive strain. This significant shift suggests that substantial compressive strain induces more than just a quantitative change in bonding strength; it qualitatively alters the orbital overlap landscape. The differential charge density plots provide spatial visualization of this altered bonding (Figure 6c,d). Under the high compressive strain (Figure 6d), the charge density difference (red circled regions) clearly shows an enhanced accumulation of electron density between the adsorbed CO and neighboring surface Pt atoms (beyond the primary adsorption site). This emergent charge accumulation signifies the formation of new or significantly strengthened bonding pathways involving adjacent Pt sites, driven by the lattice compression. This multi-site interaction explains the qualitative change in bonding observed in the COHP and underpins the deviation from the simple d-band center model under large compressive strain.

Moreover, the persistence of Pt surface segregation under experimental conditions warrants critical examination. Multiple studies demonstrate that ambient gases (e.g., O_2_, CO) significantly alter surface segregation in bimetallic systems. In the Cu-Ni system, spontaneous Cu segregation to surfaces is observed under vacuum, whereas O_2_ atmosphere induces Ni segregation from subsurface layers, which intensifies with increasing O_2_ concentration [41,42]. Similarly, the Pt-Ag system exhibits Pt enrichment in subsurface regions under vacuum, but CO atmosphere triggers Pt segregation to surface layers [43]. For the Pt-Fe system, research indicates that the extreme high O_2_ atmosphere (e.g., 1 ML O_2_) promotes the Fe surface segregation due to the strong Fe-O bonding [37]. However, such extreme conditions rarely occur in the practical catalysis. Conversely, CO preferentially adsorbs onto the exposed Pt sites with weakened affinity for Fe, thereby stabilizing the Pt terminated surface. Thus, under realistic CO-containing environments, Pt surface segregation is expected to persist, enhancing catalyst stability.

## 4. Conclusions

This study establishes a cross-scale computational framework by integrating density functional theory, cluster expansion and Monte Carlo simulations (DFT-CE-MC) to resolve the microstructure evolution and anti-CO poisoning mechanisms of Pt-Fe alloy catalysts. Our simulations predict the thermodynamic stabilization of a periodic Pt_2_Fe surface phase at ~24% Pt content, accompanied by intrinsic defect formation and subsurface Pt redistribution. DFT analysis reveals this phase intrinsically lowers the Pt d-band center (down to −2.78 eV in defect-free models) through Fe-rich coordination, weakening CO adsorption in alignment with d-band theory. Crucially, defect-induced strain disrupts the linear d-band center/adsorption relationship by triggering orbital rehybridization under compression, as evidenced by COHP and charge density analyses showing emergent multi-site Pt-CO bonding. Therefore, preserving the Pt_2_Fe surface phase while minimizing defect density maximizes CO poisoning resistance by leveraging its intrinsic electronic advantage while mitigating strain-enhanced adsorption. The DFT-CE-MC methodology demonstrated here bridges atomic-scale energetics, mesoscale structural evolution and electronic properties, providing a generalizable platform for the design of defect-engineered, CO-tolerant electrocatalysts.

## Data Availability

Data are contained within the article.
